# Supplementary material for: γ‐Secretase inhibitors in cancer clinical trials are pharmacologically and functionally distinct
Source: EMBO Mol Med. 2017 May 24;9(7):950–66. doi: 10.15252/emmm.201607265 (PMC5494507; doi:10.15252/emmm.201607265)
Supplement: Supplementary file 1 — Appendix [file EMMM-9-950-s001.pdf]

Appendix PDF

Table of Contents

Table S1	Page 2	Reported IC50s (nM) of GSIs with references.
Figure S1	Page 3	BMS-708163 inhibits rC100sub and rNOTCH1sub equivalently.
Figure S2	Page 4	WB of H4 cells stably overexpressing chimeric NOTCH1-4 and C100.
Figure S3	Page 5	The low dose potentiation of cleavage of rNOTCH3sub and rNOTCH4sub.
Figure S4	Page 6	Mammospheres growth inhibition by different GSIs at 1 and 5 μM GSIs
Figure S5	Page 7	Protein sequence of recombinant substrates used for in vitro assay.
Figure S6	Page 8	Protein sequence of substrates used for cell based assay.

Appendix Table S1. Reported IC50s (nM) of GSIs with references.

	Aβ40	NOTCH	reference
BMS-906024	1.6	0.7-3.4	(Gavai et al, 2015)
DAPT	20		(Dovey et al, 2001)
MK-0752	5	55	(Cook et al, 2010; Krop et al, 2012)
PF-3084014	6.2	13.3	(Wei et al, 2010)
RO4929097	14	5	(Luistro et al, 2009)
Semagacestat	12.1	14.1	(Mitani et al, 2012)

REFERENCES

Cook JJ, Wildsmith KR, Gilberto DB, Holahan MA, Kinney GG, Mathers PD, Michener MS, Price EA, Shearman MS, Simon AJ, Wang JX, Wu G, Yarasheski KE, Bateman RJ (2010) Acute gamma-secretase inhibition of nonhuman primate CNS shifts amyloid precursor protein (APP) metabolism from amyloid-beta production to alternative APP fragments without amyloid-beta rebound. *J Neurosci* **30:** 6743-6750

Dovey HF, John V, Anderson JP, Chen LZ, de Saint Andrieu P, Fang LY, Freedman SB, Folmer B, Goldbach E, Holsztynska EJ, Hu KL, Johnson-Wood KL, Kennedy SL, Kholodenko D, Knops JE, Latimer LH, Lee M, Liao Z, Lieberburg IM, Motter RN et al (2001) Functional gamma-secretase inhibitors reduce beta-amyloid peptide levels in brain. *J Neurochem* **76:** 173-181

Gavai AV, Quesnelle C, Norris D, Han WC, Gill P, Shan W, Balog A, Chen K, Tebben A, Rampulla R, Wu DR, Zhang Y, Mathur A, White R, Rose A, Wang H, Yang Z, Ranasinghe A, D'Arienzo C, Guarino V et al (2015) Discovery of Clinical Candidate BMS-906024: A Potent Pan-Notch Inhibitor for the Treatment of Leukemia and Solid Tumors. *ACS medicinal chemistry letters* **6:** 523-527

Krop I, Demuth T, Guthrie T, Wen PY, Mason WP, Chinnaiyan P, Butowski N, Groves MD, Kesari S, Freedman SJ, Blackman S, Watters J, Loboda A, Podtelezhnikov A, Lunceford J, Chen C, Giannotti M, Hing J, Beckman R, Lorusso P (2012) Phase I pharmacologic and pharmacodynamic study of the gamma secretase (Notch) inhibitor MK-0752 in adult patients with advanced solid tumors. *Journal of clinical oncology : official journal of the American Society of Clinical Oncology* **30:** 2307-2313

Luistro L, He W, Smith M, Packman K, Vilenchik M, Carvajal D, Roberts J, Cai J, Berkofsky-Fessler W, Hilton H, Linn M, Flohr A, Jakob-Rotne R, Jacobsen H, Glenn K, Heimbrook D, Boylan JF (2009) Preclinical profile of a potent gamma-secretase inhibitor targeting notch signaling with in vivo efficacy and pharmacodynamic properties. *Cancer Res* **69:** 7672-7680

Mitani Y, Yarimizu J, Saita K, Uchino H, Akashiba H, Shitaka Y, Ni K, Matsuoka N (2012) Differential effects between gamma-secretase inhibitors and modulators on cognitive function in amyloid precursor protein-transgenic and nontransgenic mice. *J Neurosci* **32:** 2037-2050

Wei P, Walls M, Qiu M, Ding R, Denlinger RH, Wong A, Tsaparikos K, Jani JP, Hosea N, Sands M, Randolph S, Smeal T (2010) Evaluation of selective gamma-secretase inhibitor PF-03084014 for its antitumor efficacy and gastrointestinal safety to guide optimal clinical trial design. *Molecular cancer therapeutics* **9:** 1618-1628

Appendix Figure S1. BMS-708163 inhibits rC100sub and rNOTCH1sub equivalently.

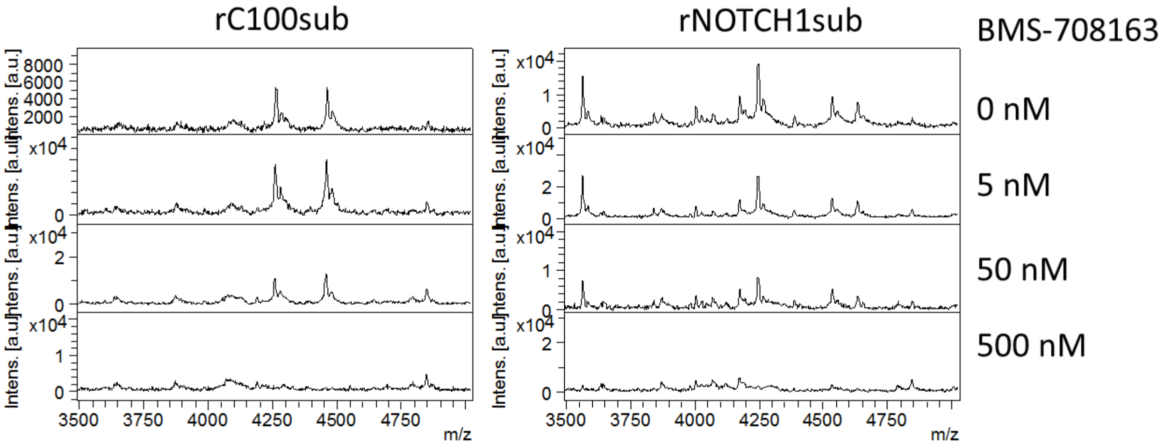

Appendix Figure S2. WB of H4 cells stably overexpressing chimeric NOTCH1-4 and C100. Detected with 6E10 antibody.

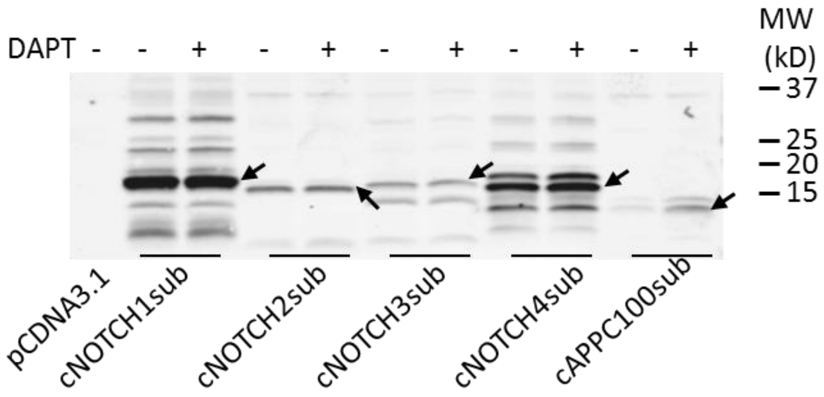

Appendix Figure S3. The low dose potentiation of cleavage of rNOTCH3sub and rNOTCH4sub. CHAPSO solubilized CHO membrane was incubated with rNOTCH3sub and rNOTCH4sub. The Aβ-Nβ chimeric peptides with different concentration of BMS-906024 and Semagacestat were compared using IP-MS. G1456 from rNOTCH4sub and a peak about 3100 Da from rNOTCH3sub were used as internal-standards as shown on the left side of each spectrum.

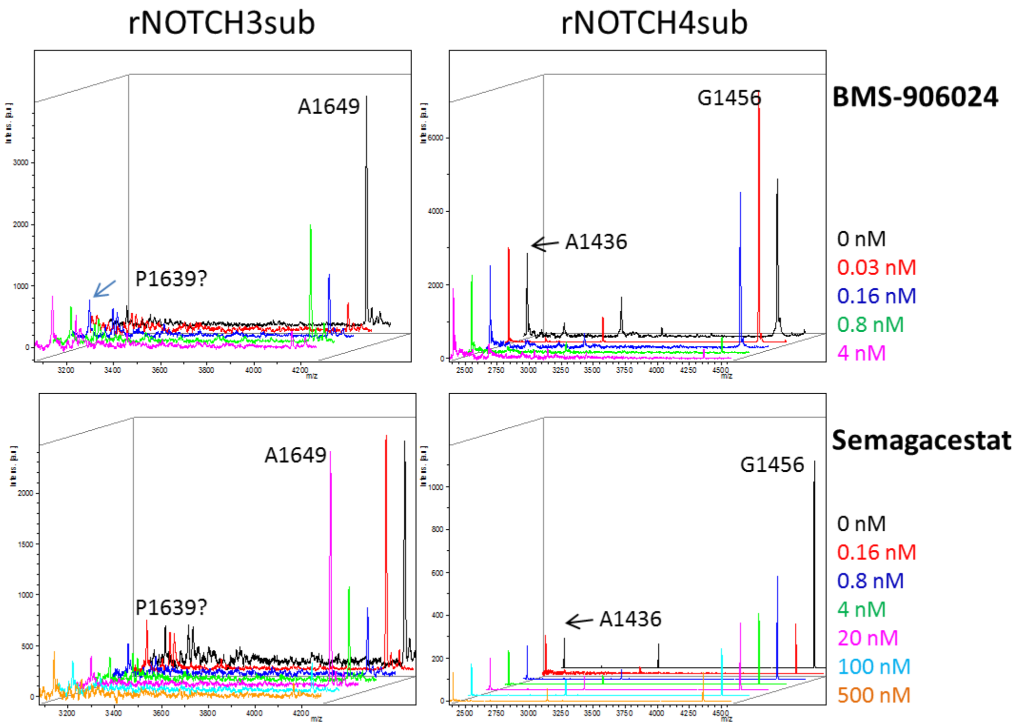

Appendix Figure S4. Mammospheres growth inhibition by different GSIs at 1 and 5 μM GSIs. A. Limiting dilution assays of MDA-MB-231. B. Limiting dilution assays of MDA-MB-468 cell lines. Large mammospheres (≥500 μm in diameter) and small mammospheres (100-500 μm) of 10 μM GSIs treated samples were counted. C. Absolute cell counts from the limiting dilution assay at of MDA-MB-231. D. Absolute cell counts from the limiting dilution assay at of MDA-MB-468. All data were analyzed with one-way ANOVA using GraphPad Prism6 software (*p<0.05, **p<0.01, ***p<0.001, ****p<0.0001).

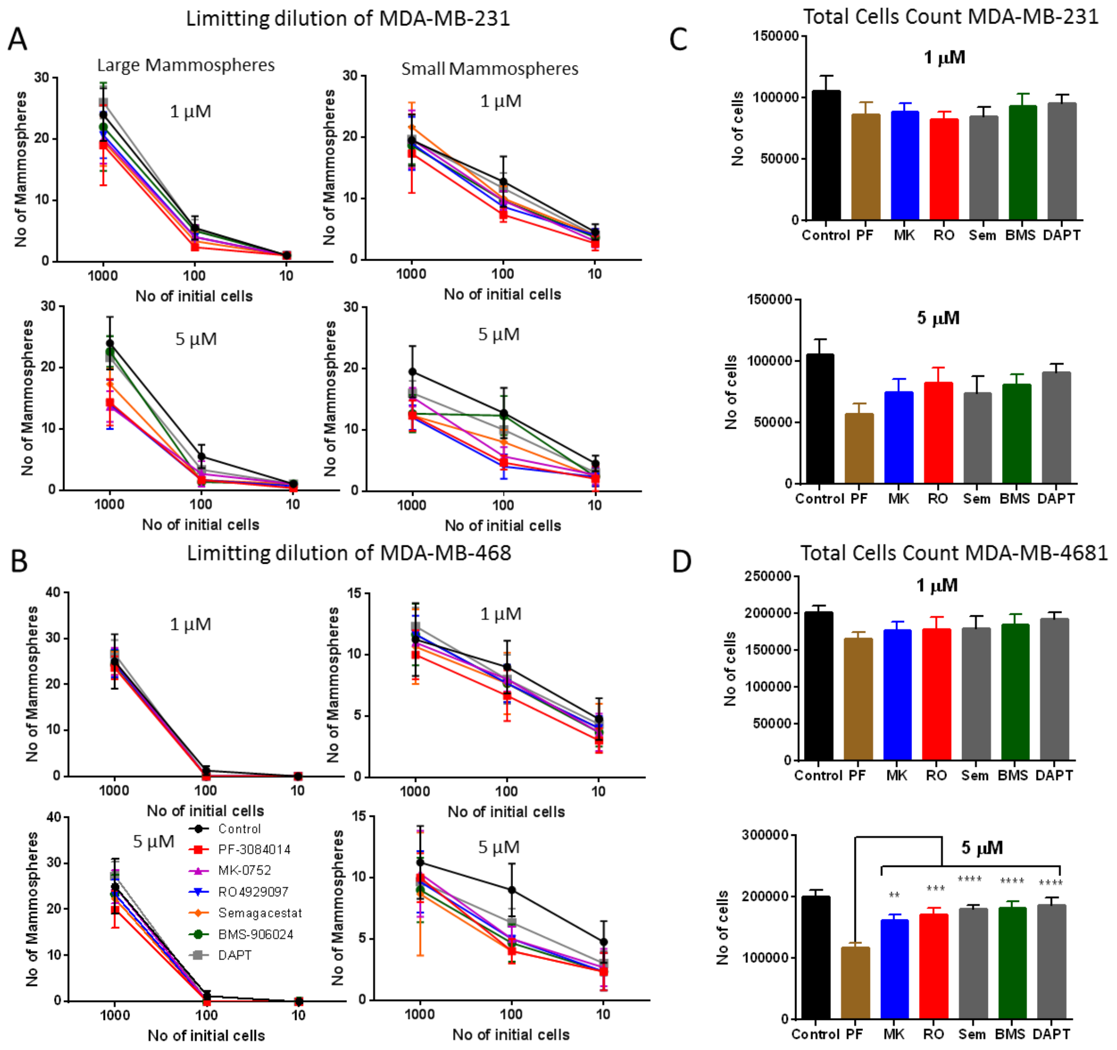

Appendix Figure S5. Protein sequence of recombinant substrates used for in vitro assay. Red, $A\beta_{1-15}$. Black, NOTCH fragment. Green, FLAG tag. Underlined, predicted transmembrane domain.

rNOTCH1sub:
MDAEFRHDSGYEVHHQVQSETVEPPPPAQLHFMYVAAAAFVLLFFVGCGVLLSRKRRRQHGQLWFPEGFKVSEASKKKRREPLGED
SVGLKPLKNASDGALMDDNQNEWGDEDLEDYKDDDDK

rNOTCH2sub:
MDAEFRHDSGYEVHHQVVSESLTPERTQLLYLLAVAVVIILFIILLGVIMAKRKRKHGSLWLPEGFTLRRDASNHKRREPVGQDAV
GLKNLSVQVSEANLIGTGTSEHWVDDEGPDYKDDDDK

rNOTCH3sub:
MDAEFRHDSGYEVHHQVRGEPLEPPEPSVPLLPLLVAGAVLLLVILVLGVMVARRKREHSTLWFPEGFSLHKDVASGHKGRREPVG
QDALGMKNMAKGESLMGEVATDWMDTECPDYKDDDDK

rNOTCHsub4:
MDAEFRHDSGYEVHHQHPHAGTAPPANQLPWPVLCSPVAGVILLALGALLVLQLIRRRRREHGALWLPPGFTRRPRTQSAPHRRRP
PLGEDSIGLKALKPKAEVDEDGVVMCSGPDYKDDDDK

rCD44sub:
MDAEFRHDSGYEVHHQNTTSGPIRTPQIPEWLIILASLLALALILAVCIAVNSRRRCGQKKKLVINSGNGAVEDRKPSGLNGEASK
SQEMVHLVNKESSETPDQFMTADETRNLQNVDMKIGVDYKDDDDK

rVEGFR1sub:
MDAEFRHDSGYEVHHQSAYLTVQGTSDKSNLELITLTCTCVAATLFWLLLTLFIRKMKRSSSEIKTDYLSIIMDPDEVPLDEQCER
LPYDASKWEFARERLKLGKSLGRGAFGKVDYKDDDDK

Appendix Figure S6. Protein sequence of substrates used for cell based assay. Blue, Signal peptide from NOTCH 4. Red, A$\beta_{1\text{-}25/K16A}$. Black, NOTCH fragment. Green, FLAG tag. Underlined, predicted transmembrane domain.

cNOTCH1sub:
MDPPSLLLLLLLLLLLCVSVVRPRGDAEFRHDSGYEVHHQALVFFAEDVGVQSETVEPPPPAQLHFMYVAAAAFVLLFFVGCGVLL
SRKRRRQHGQLWFPEGFKVSEASKKKRREPLGEDSVGLKPLKNASDGALMDDNQNEWGDEDLEDYKDDDDK

cNOTCH2sub:
MDPPSLLLLLLLLLLLCVSVVRPRGDAEFRHDSGYEVHHQALVFFAEDVGVVSESLTPERTQLLYLLAVAVVIILFIILLGVIMAK
RKRKHGSLWLPEGFTLRRDASNHKRREPVGQDAVGLKNLSVQVSEANLIGTGTSEHWVDDEGPDYKDDDDK

cNOTCH3sub:
MDPPSLLLLLLLLLLLCVSVVRPRGDAEFRHDSGYEVHHQALVFFAEDVGVRGEPLEPPEPSVPLLPLLVAGAVLLLVILVLGVMV
ARRKREHSTLWFPEGFSLHKDVASGHKGRREPVGQDALGMKNMAKGESLMGEVATDWMDTECPDYKDDDDK

cNOTCH4sub:
MDPPSLLLLLLLLLLLCVSVVRPRGDAEFRHDSGYEVHHQALVFFAEDVGHPHAGTAPPANQLPWPVLCSPVAGVILLALGALLVL
QLIRRRRREHGALWLPPGFTRRPRTQSAPHRRRPPLGEDSIGLKALKPKAEVDEDGVVMCSGPDYKDDDDK

cCD44sub:

MDPPSLLLLLLLLLLLCVSVVRPRGDAEFRHDSGYEVHHQALVFFAEDVGNTTSGPIRTPQIPEWLIILASLLALALILAVCIAVN
SRRRCGQKKKLVINSGNGAVEDRKPSGLNGEASKSQEMVHLVNKESSETPDQFMTADETRNLQNVDMKIGVDYKDDDDK

cVEGFR1sub:
MDPPSLLLLLLLLLLLCVSVVRPRGDAEFRHDSGYEVHHQALVFFAEDVGSAYLTVQGTSDKSNLELITLTCTCVAATLFWLLLTL
FIRKMKRSSSEIKTDYLSIIMDPDEVPLDEQCERLPYDASKWEFARERLKLGKSLGRGAFGKVDYKDDDDK
