# Supplementary material for: γ‐Secretase inhibitors in cancer clinical trials are pharmacologically and functionally distinct
Source: EMBO Mol Med. 2017 May 24;9(7):950–66. doi: 10.15252/emmm.201607265 (PMC5494507; doi:10.15252/emmm.201607265)
Supplement: Supplementary file 3 — Source Data for Figure 6 [file EMMM-9-950-s002.pptx]

## Slide 1
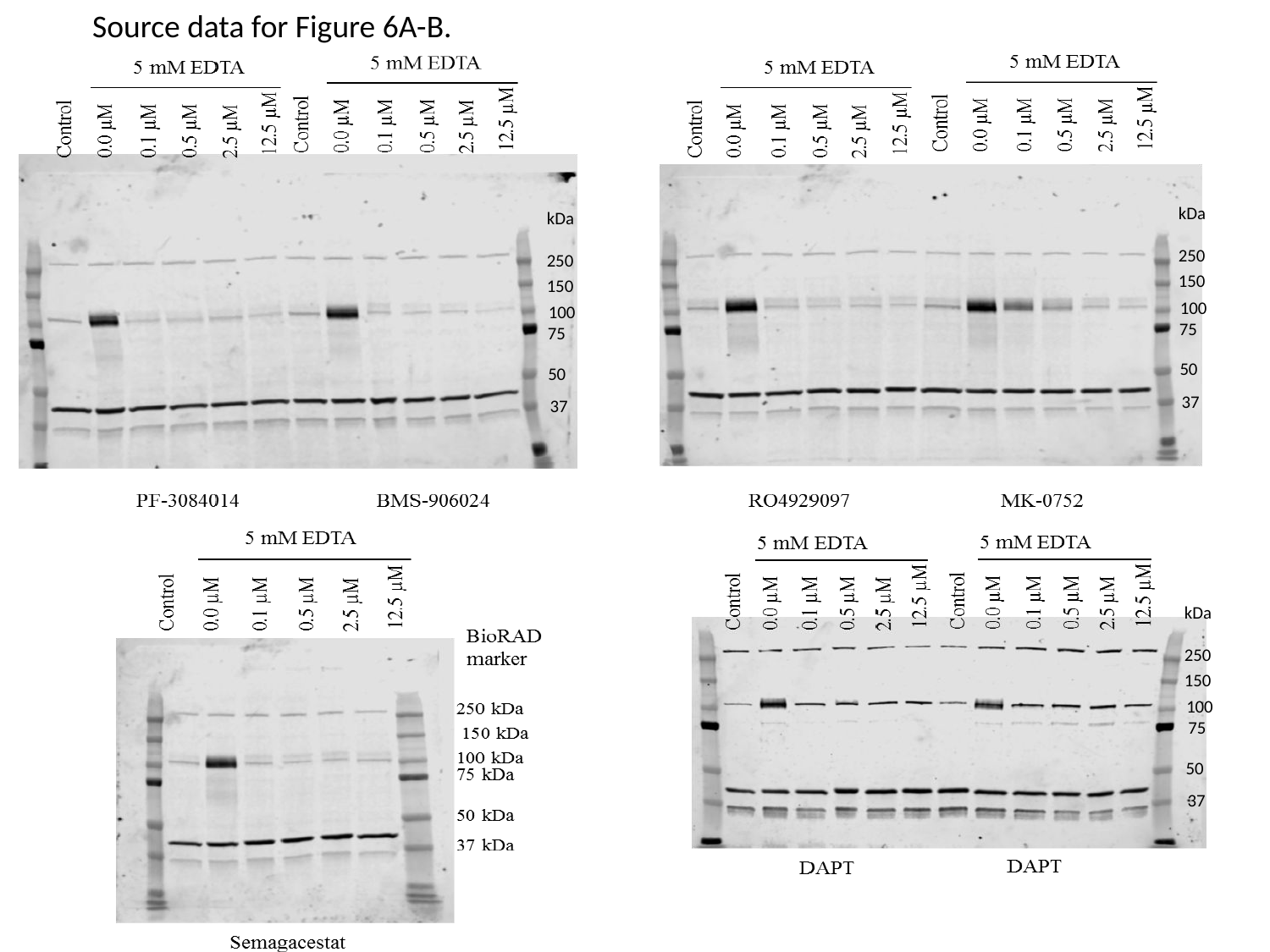

Source data for Figure 6A-B.
kDa
250
kDa
250
150
150
100
100
75
75
50
50
37
37
kDa
250
150
100
75
50
37

## Slide 2
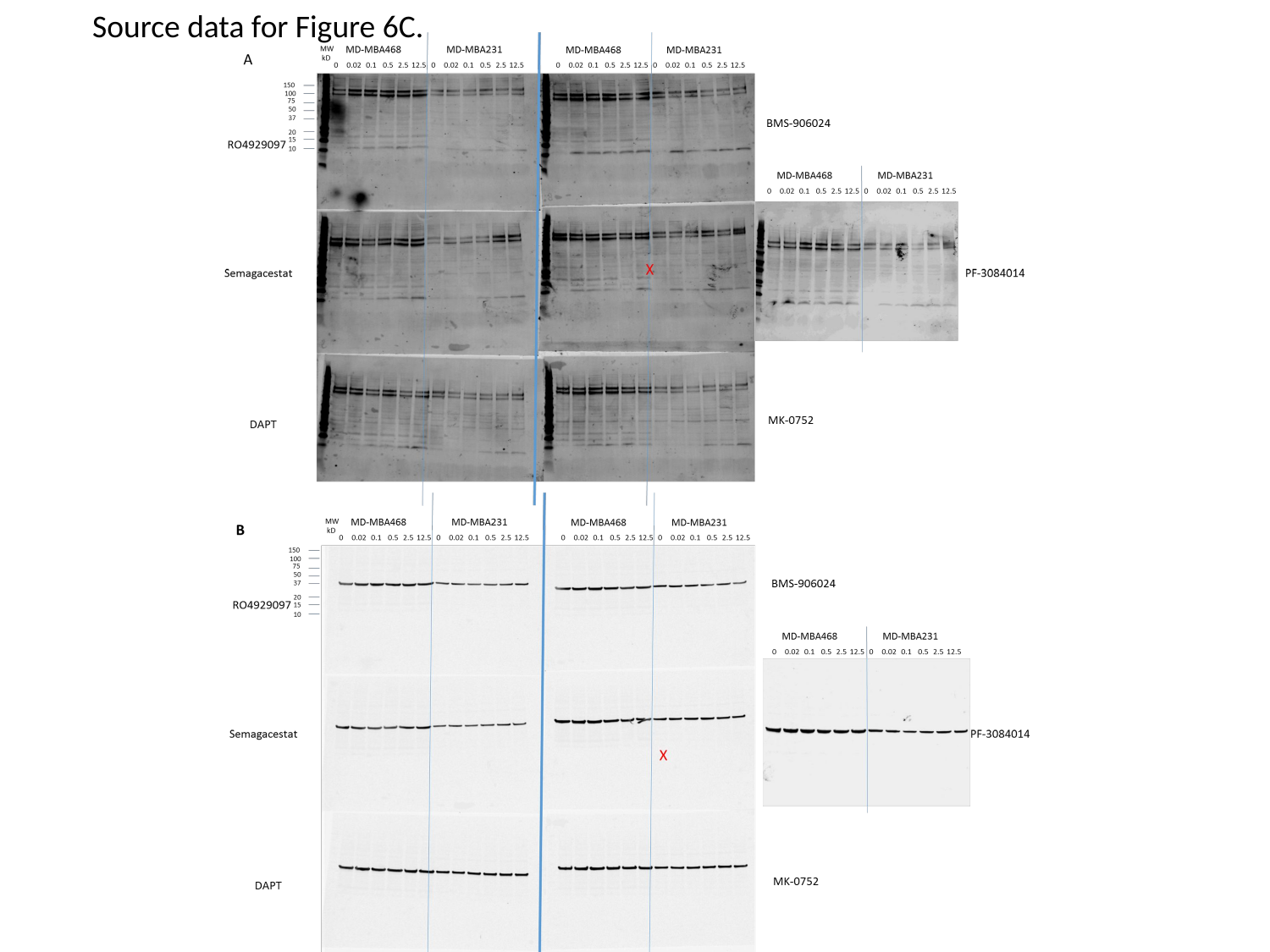

Source data for Figure 6C.

## Slide 3
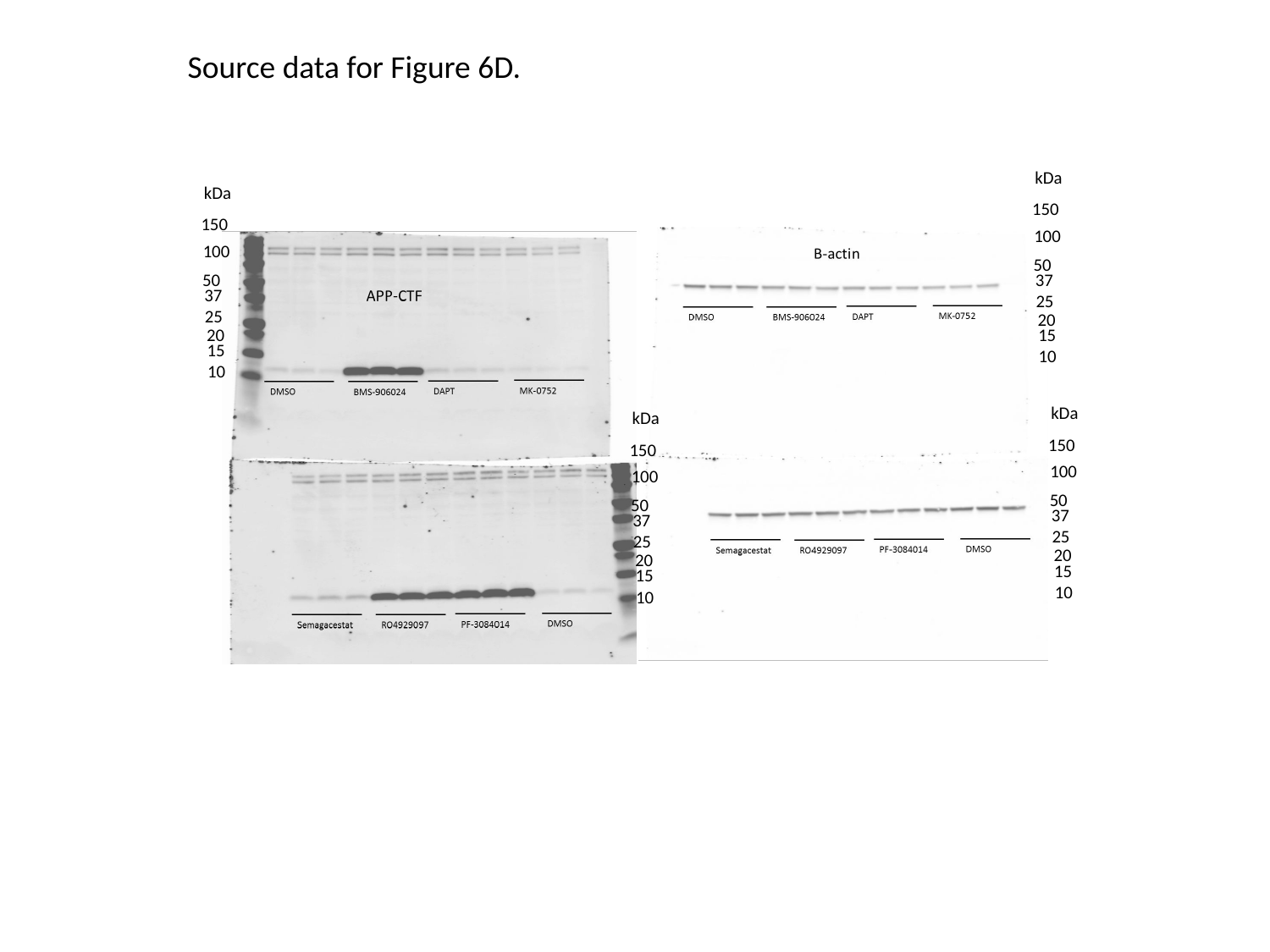

Source data for Figure 6D.
kDa
kDa
150
150
100
100
50
50
37
37
25
25
20
20
15
15
10
10
kDa
kDa
150
150
100
100
50
50
37
37
25
25
20
20
15
15
10
10
