# Supplementary figures and images for: γ‐Secretase inhibitors in cancer clinical trials are pharmacologically and functionally distinct
Source: EMBO Mol Med. 2017 May 24;9(7):950–66. doi: 10.15252/emmm.201607265 (PMC5494507; doi:10.15252/emmm.201607265)

Source data for Figure 8C.

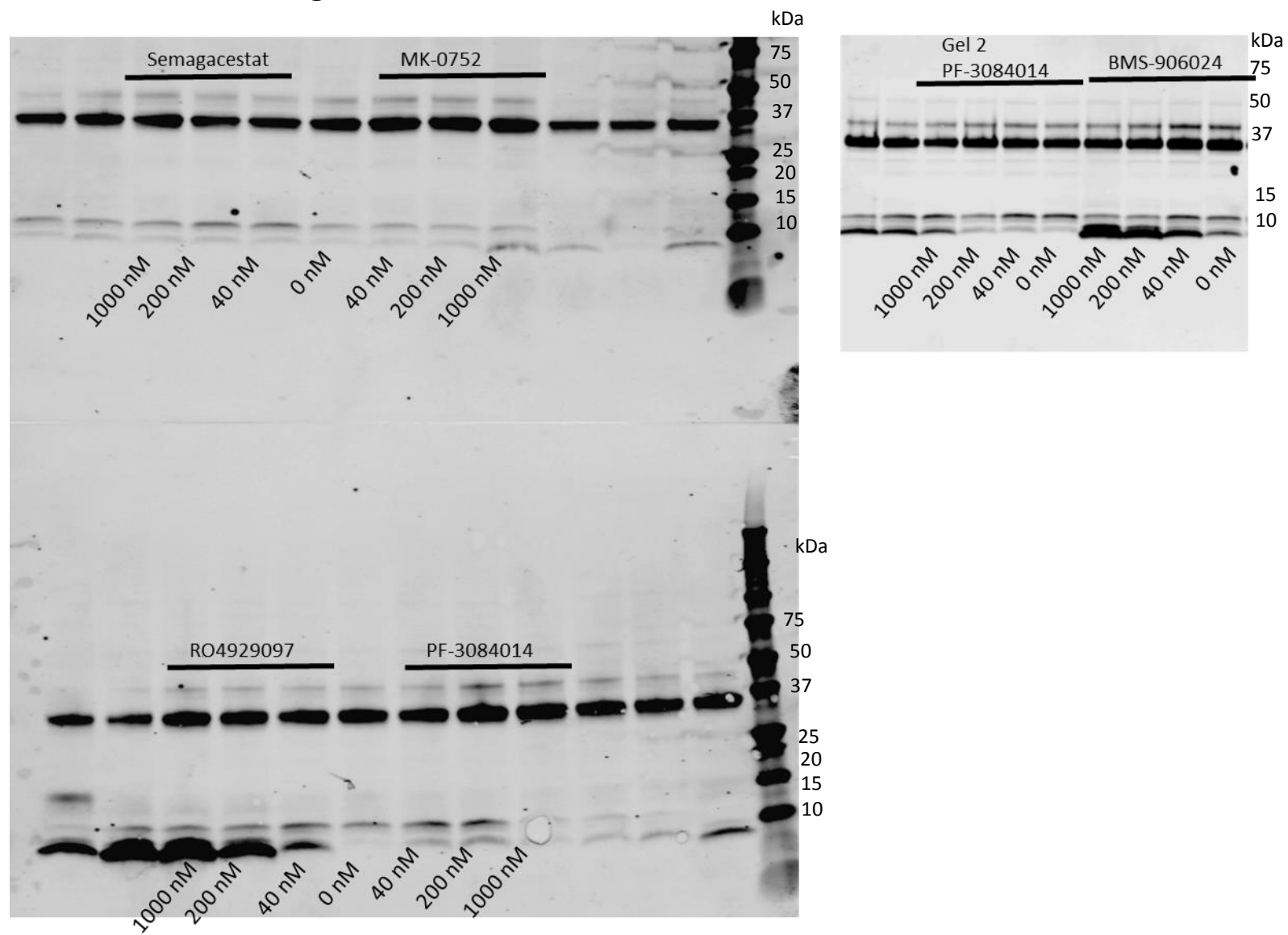

Supplement: Supplementary file 4 — Source Data for Figure 8 [file EMMM-9-950-s003.pdf]
